# Sources and atmospheric transformation of PM_2.5_ at forest site in southern China revealed by δ^13^C

**DOI:** 10.1016/j.isci.2026.116574

**Published:** 2026-06-29

**Authors:** Zhenyu Cheng, Qing Man, Jiaoping Xing, Jiahui Peng, Runping Ding, Feifeng Chen, Wenhua Wang, Qing Ye, Yuanqiu Liu, Yan Wang, Linping Zhang

**Affiliations:** 1 Jiangxi Provincial Key Laboratory of Conservation Biology, School of Forestry, Jiangxi Agricultural University, Nanchang 330045, China; 2 Key Laboratory of State Forestry and Grassland Administration on Forest Ecosystem Protection and Restoration of Poyang Lake Watershed, School of Forestry, Jiangxi Agricultural University, Nanchang 330045, China; 3 School of Resources and Materials, Northeastern University at Qinhuangdao, Qinhuangdao 066004, China

**Keywords:** chemical compositions, source apportionment, stable carbon isotopic composition, aging process, carbonaceous aerosols

## Abstract

Suburban forest sites are influenced by both biogenic emissions and anthropogenic pollutants from nearby urban areas. This study investigated PM_2.5_ sources and atmospheric transformation processes using chemical components and stable carbon isotopes (δ^13^C) at a suburban forest site in southern China. The average PM_2.5_ concentration was 62.72 ± 28.90 μg/m^3^, dominated by secondary inorganic aerosols and organic matter. SOC contributed about 53% of OC during the sampling period. PMF identified six major sources, including biomass burning, vehicle emissions, coal combustion, dust, industrial dust, and waste incineration. PSCF results showed that PM_2.5_ and OC were affected by both local emissions and regional transport in autumn, but mainly by local sources in winter. δ^13^C analysis indicated that carbonaceous aerosols were primarily derived from C3 plant biomass and vehicular emissions. High WSOC/OC and SOC/OC ratios suggested significant aerosol aging and secondary organic carbon formation, which strongly influenced δ^13^C variations in PM_2.5_.

## Highlights

• Carbonaceous aerosols mainly derived from vehicle exhaust and biomass burning (C3 plants)
• Lower δ^13^C_PM2.5_ in winter is attributed to SOC formation
• Higher δ^13^C_PM2.5_ in autumn was due to aging and secondary formation processes

## Graphical abstract

**Figure:**
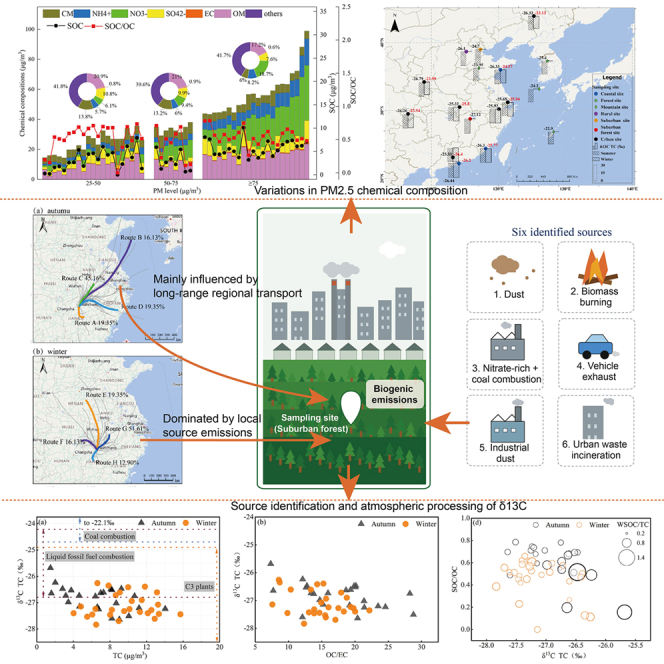

## Teaser

earth sciences; environmental science; environmental issues.

## Introduction

Haze pollution is a major global environmental issue with negative effects on public health,^1^ air visibility,^2^ and climate.^3^ Rapid urbanization and industrialization in China in recent decades have resulted in frequent large-scale haze events, primarily due to elevated concentrations of PM_2.5_.^4^ Even though annual PM_2.5_ concentrations have declined after implementing emission control measures,^4^^,^^5^ exceedances of the National Ambient Air Quality Standards (NAAQSs) remain frequent, particularly in winter.^6^ Thus, developing a comprehensive comprehension of PM_2.5_ sources and atmospheric processes is essential for implementing effective pollution mitigation and prevention strategies.

Numerous field studies have been carried out to investigate the chemical composition, emission sources, and formation mechanisms of PM_2.5_ during intense haze events.^7^^,^^8^^,^^9^ Fine particulate matter is a chemically complex assemblage composed primarily of secondary inorganic aerosols, carbonaceous species, trace elements, and mineral dust.^10^ Recent evidence indicates that under heavy pollution conditions, PM_2.5_ is primarily composed of secondary constituents, including secondary inorganic species i.e., sulfate, nitrate, and ammonium, together with secondary organic aerosols (SOCs) produced via atmospheric oxidation processes involving volatile organic compounds (VOCs).^11^

Source apportionment analyses have identified major contributors to PM_2.5_ as secondary aerosol formation, motor vehicle exhaust, coal burning, dust, biomass combustion, and industrial activities.^12^ Based on positive matrix factorization (PMF) results from the sampling site at Yudu Park in southern Shanxi Province, China, the contributions of coal combustion and biomass burning to ambient PM_2.5_ have decreased over the past decade, whereas the contribution of secondary aerosols has increased.^8^ The development and temporal evolution of haze pollution involve a series of complex atmospheric processes, including aerosol aging, fog and cloud processing, primary emissions, and secondary chemical production.^13^ Nevertheless, most existing studies on PM_2.5_ characteristics, sources, and studies on their formation mechanisms in China have mainly concentrated on urban areas.^14^^,^^15^ As urban emissions have been substantially reduced in recent years, emissions originating from rural and suburban regions are becoming increasingly important for effective air quality management.

Previous studies have found that carbonaceous components constitute roughly 20%–50% of total PM_2.5_ mass and may make up as much as 90% in forest regions.^16^ Being a primary constituent of PM_2.5_, carbonaceous constituents are commonly classified into organic carbon (OC) and elemental carbon (EC), with OC further consisting of primary OC (POC) and SOC. SOC mainly forms via photochemical oxidation of gaseous precursors, including VOCs. Currently, source apportionment of carbonaceous components in PM_2.5_ is conducted using a variety of approaches, including the OC/EC ratio method,^17^ compositional analysis (e.g., principal component analysis),^18^ carbon isotope analysis (^14^C and ^13^C),^19^ PMF, and backward trajectory clustering analysis (HYSPLIT), among others. For instance, δ^13^C values of PM_1_ in Mexico varied between −27.0‰ and −15.0‰, indicating dominant contributions from C3 and C4 plant sources.^20^ Similarly, δ^13^C measurements of PM_2.5_ in northern cities of China were −24.4‰ ± 0.8‰ in winter and −26.5‰ ± 0.2‰ in summer on average, suggesting that carbonaceous aerosols primarily originated from coal combustion and vehicular emissions, with a stronger influence of coal combustion during wintertime.^21^ Suburban forests play an important role in carbon fixation and storage,^22^ provide habitats for various species and support urban biodiversity,^23^ and regulate energy and water fluxes to mitigate the urban heat island effect, thereby influencing regional climate.^24^ In addition, suburban forests can affect air quality through multiple pathways.^25^ For example, by removing atmospheric pollutants and improving air quality, meanwhile, the emission of VOCs from suburban forest participates in atmospheric chemical processes, contributing to the formation of ozone (O_3_) and fine particulate matter (PM_2.5_).^26^ As a transitional zone between urban and forest environments, suburban forest sites exhibit distinct particulate matter formation mechanisms compared to those at purely urban, suburban, or forest sites, as they are influenced by both biogenic emissions and anthropogenic pollutants transported from surrounding cities. Also, forest regions affected by anthropogenic air masses enriched in NO_X_ are expected to enhance SOC formation. Moreover, forest environments often have relatively high humidity, in which conditions biogenic SOC and primary biological aerosol particles can contribute substantially to the submicron WSOC fraction.^27^ Therefore, improved understanding of chemical processing dynamics in suburban forest environments and their interactions with urban atmospheric systems is essential.^28^

**Figure 1:**
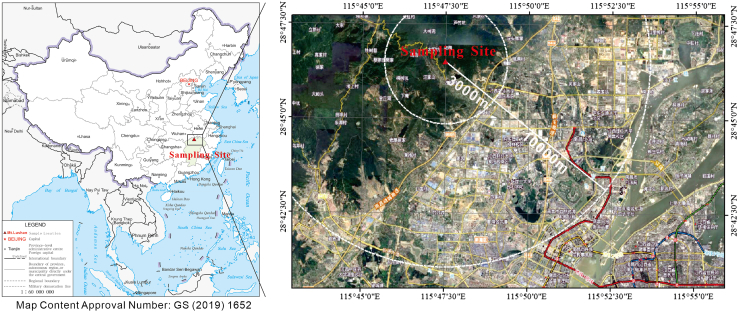
Map of the study area showing the sampling site

This study presents an integrated dataset of chemical species and stable carbon isotope measurements in ambient aerosols collected at a suburban forest site in southern China (Figure 1). The specific objectives are to (1) characterize the principal chemical composition of PM_2.5_ at the suburban forest site; (2) investigate the δ^13^C_OC_, δ^13^C_EC_, and δ^13^C_TC_ characteristics; (3) identify the potential sources of PM_2.5_ and its carbonaceous fractions; and (4) elucidate the chemical processing dynamics in suburban forest environments and their interactions with the surrounding urban atmosphere. Overall, this work advances the understanding of aerosol properties, source contributions, and evolutionary processes in suburban forest regions of southern China.

## Results and discussion

### Chemical compositions in PM_2.5_ and their trends at different pollution conditions

During the sampling campaign, daily PM_2.5_ concentrations at the suburban forest site varied between 31.14 and 147.16 μg/m^3^, with a mean value of 62.72 ± 28.90 μg/m^3^. Seasonal analysis indicated higher PM_2.5_ levels in winter (88.14 ± 24.06 μg/m^3^) than in autumn (46.87 ± 14.90 μg/m^3^). The mean daily PM_2.5_ level in winter surpassed the NAAQS of 75 μg/m^3^, suggesting that the suburban forest site experienced moderate pollution during the winter period. The results show that 41.2% of the samples were within the range of 25–50 μg/m^3^, 15.7% were within 50–75 μg/m^3^, and 43.1% exceeded the NAAQS limit (≥75 μg/m^3^). The relatively high exceedance frequency indicates that pollution events occurred frequently at this suburban forest site, especially during winter.

Research indicates that the primary chemical constituents of PM_2.5_ in urban regions of China are secondary inorganic aerosols (NH_4_^+^ + SO_4_^2−^ + NO_3_^−^), total carbonaceous (OM and EC) and fine soil or crustal materials (CMs).^29^^,^^30^ To assess the representativeness of the measured chemical components, PM_2.5_ mass was reconstructed using these species.^14^ In the present study, the OC to OM conversion factor was set to 1.6.^12^ CM concentrations were calculated following Yao et al.^31^ as [Crustal materials] = 2.20 ∗ [Al] + 2.49 ∗ [Si] + 1.63 ∗ [Ca] + 1.42 ∗ [Fe] + 1.94 ∗ [Ti]. As silicon was not measured in this study, the median Si/Al ratio of 3.54 in east Asian deserts was used.^32^ It should be noted that the study area is located in the subtropical forests of southern China. Despite the fact that its Si/Al ratio is lower than that of dust source regions, which may result in some estimation bias, it remains within a reasonable range of variation.^33^

There is a strong correlation between measured and reconstructed PM_2.5_ mass concentrations (R^2^ > 0.90), with an average slope of 0.63, indicating that SIA, total carbonaceous aerosols (TCAs), and CM largely represented the PM_2.5_ composition at the suburban forest site. Previous studies have shown that the mass of PM_2.5_ reconstructed through chemical composition typically accounts for only 60%–80% of the total mass, with the remainder is primarily attributed to aerosol water, unmeasured organic matter (OM), and crusting material.^34^^,^^35^

**Figure 2:**
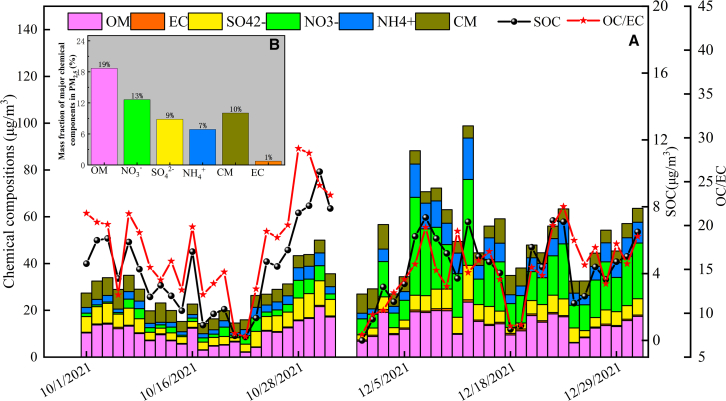
Temporal trends and chemical composition of PM_2.5_ during the sampling period (A) Temporal variations in PM_2.5_ chemical composition, SOC concentrations, and OC/EC ratios during the sampling period. (B) Average mass fractions of the major chemical components in PM_2.5_.

On average, SIA, TCA, and CM accounted for 29% ± 9%, 20% ± 6%, and 10% ± 4% of PM_2.5_ mass, respectively (Figure 2). Among individual species, OM contributed the highest fraction (19% ± 6%), exceeding NO_3_^−^ (13% ± 8%), SO_4_^2−^ (9% ± 4%), CM (10% ± 4%), NH_4_^+^ (7% ± 2%), and EC (1% ± 0.1%). Peak OM levels reached 34.4%. Note that the conversion factor from OC to OM was slightly higher for aged OC (2.1 ± 0.2) than for urban aerosol (1.6 ± 0.2).^34^ If using the conversion factor of 2.1 ± 0.2 instead of 1.6 ± 0.2, the mass fractions of OM in PM_2.5_ would reach 39% during the sampling time, and the reconstructed PM_2.5_ mass would account for 80%. OM is composed of POC and SOC, the latter being produced through photochemical oxidation of gaseous precursors, such as VOCs. While the POC is mainly attributed to coal combustion, biomass burning, and vehicle exhaust.

SOC was estimated using the OC/EC ratio and the EC tracer method,^36^ where OC/EC > 2.0 indicates SOC formation.^37^ In this study, OC/EC ratios ranged from 6.19 to 28.79, with an average of 16.08, suggesting significant SOC formation in the suburban forest, and substantially higher than ratios reported in nearby urban areas.^38^ The (OC/EC)_pri_ ratio was determined using the MRS method, which reduces uncertainty associated with empirical assumptions and improves the robustness of SOC estimation. Estimated SOC concentrations, based on the EC tracer method,^39^ ranged from 0.20 to 10.10 μg/m^3^, averaging 4.27 μg/m^3^ across the sampling period. Seasonal averages were comparable, with 4.19 μg/m^3^ in autumn and 4.27 μg/m^3^ in winter. SOC accounted for approximately 53% of total OC during the sampling period, with seasonal contributions of 60.40% in autumn and 46.16% in winter. These results indicate that organic compounds are dominant in PM_2.5_ at this suburban forest site, with secondary OC comprising the majority of the organic fraction.

**Figure 3:**
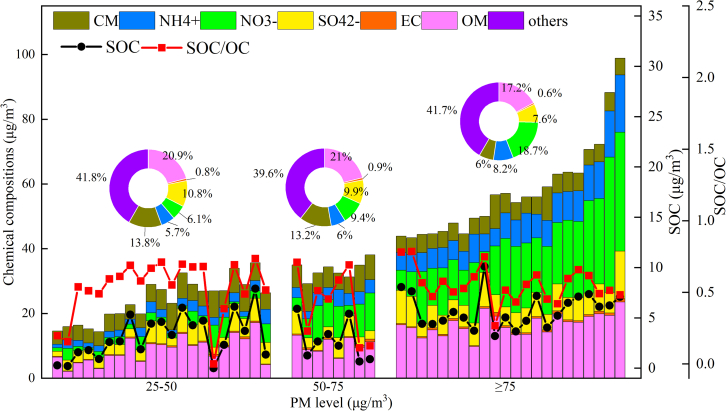
Mass fraction of major chemical components in PM_2.5_ during the sampling period (the pie charts show the average mass fractions of the corresponding chemical components for each PM_2.5_ level category)

As described above, PM_2.5_ concentrations remained consistently at relatively high levels (≥75 μg/m^3^) throughout the entire sampling period, accounting for 43.1% of the observations. Figure 3 summarizes the chemical composition characteristics of PM_2.5_ under different pollution levels, which were classified into three concentration ranges: 25–50 μg/m^3^, 50–75 μg/m^3^, and ≥75 μg/m^3^. When PM_2.5_ concentrations were 25–50 μg/m^3^, OM accounted for 20.9% of the total mass, decreasing to 17.2% when PM_2.5_ exceeded 75 μg/m^3^. With increasing PM_2.5_ levels, the mass fractions of mineral components (CM) and SO_4_^2−^ also decreased, from 13.8% to 6.0% and from 10.8% to 7.6%, respectively. In contrast, NO_3_^−^ and NH_4_^+^ showed increasing trends in PM_2.5_, rising from 6.1% to 18.7% and from 5.7% to 8.2%, respectively.

The proportion of EC showed little variation across different PM_2.5_ concentration levels, indicating relatively stable primary emissions. In contrast, SOC concentrations increased significantly with increasing PM_2.5_ levels, suggesting enhanced secondary formation under polluted conditions. A similar increasing trend was observed for NO_3_^−^. These results indicate that elevated PM_2.5_ concentrations during heavily polluted periods were mainly driven by the formation of secondary components, particularly SOC and nitrate. The proportion of nitrates increases significantly as pollution levels rise, which may be related to high NO_X_ emissions from urban areas. Studies have shown that elevated relative humidity (RH) suppresses the volatility of ammonium nitrate, enhances the efficiency of gas-to-particle conversion of nitrates, and further promotes their continuous accumulation.^40^ Additionally, a weak negative correlation between NO_2_ and SOC was observed, suggesting that NO_X_ may influence VOC oxidation at the suburban forest site. Previous studies have shown that under low-NO_X_ conditions, BVOC oxidation is primarily driven by reactions with HO_2_ radicals, leading to the formation of low-volatility products and enhanced SOA formation.^41^^,^^42^ Under high-NO_X_ conditions, however, oxidation pathways increasingly involve reactions with NO and NO_2_, producing relatively more volatile compounds and altering the chemical and isotopic characteristics of SOC.^43^^,^^44^

### Possible sources of PM_2.5_ by PMF

**Figure 4:**
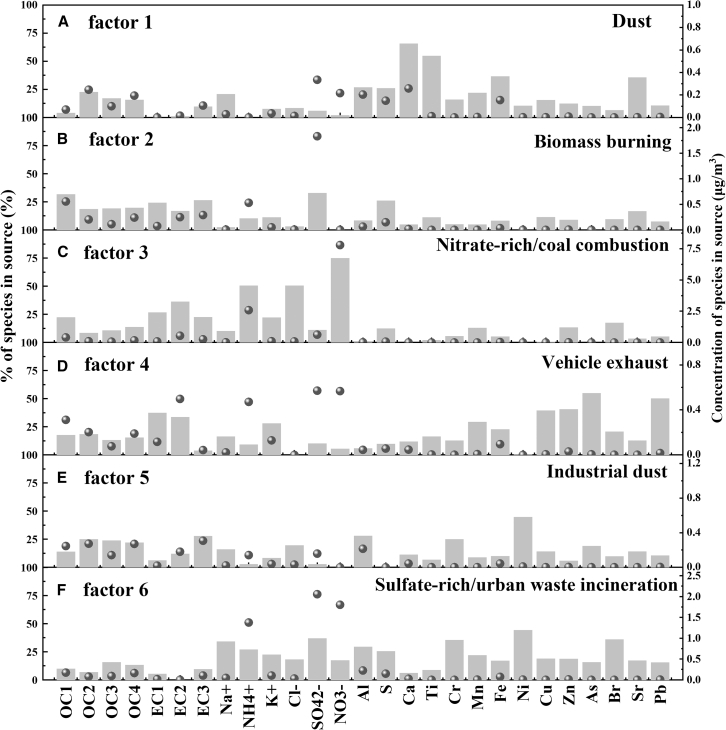
Factor profiles (μg/m^3^ and % of the total species) obtained from the PMF analysis (A) Dust, (B) biomass burning, (C) nitrate-rich/coal combustion, (D) vehicle exhaust, (E) industrial dust, and (F) sulfate-rich/urban waste incineration.

PMF was applied to identify and quantify the sources of PM_2.5_ at the suburban forest site. Daily PM_2.5_ chemical composition data, including carbonaceous components (OC1-4 and EC1-3), water-soluble ions (Na^+^, NH_4_^+^, K^+^, Cl^−^, NO_3_^−^, and SO_4_^2−^), and trace elements (Al, S, Ca, Ti, Cr, Mn, Fe, Ni, Cu, Zn, As, Br, Sr, and Pb), were incorporated into PMF 5.0 to estimate the contributions of individual sources to the total particulate mass. Various factor numbers ranging from three to twelve were tested, and a six factor solution was selected as the most representative for capturing all major sources. As illustrated in Figure 4, six sources were identified: dust, biomass burning, nitrate-rich/coal combustion, vehicle exhaust, industrial dust, and urban waste incineration. Source profiles and a brief description of each resolved factor are provided in Figure 4.

The first factor (F1) was dominated by substantial contributions from Ca (65.7%), Ti (54.8%), Fe (36.6%), Sr (35.7%), and Al (26.8%). These elements are widely recognized as typical markers of crustal or soil-related sources.^45^ Therefore, factor 1 might be dust. The second factor (F2) had high loading of OC1 (31.8%), OC2 (18.5%), OC3 (19.1%), along with moderate loadings of K^+^ (11.1%). K^+^ has usually been used as the tracer for biomass combustion in receptor models,^46^^,^^47^ while OC1 normally rich in the open burning of straw.^48^ Accordingly, this factor was attributed to biomass combustion. The third factor (F3) was mainly loaded with NO_3_^−^ (75.1%), Cl^−^ (50.6%), NH_4_^+^ (50.7%), EC2 (36.3%), EC3 (22.6%), EC1 (26.7%), and OC1 (22.5%). This factor was dominated by elevated NO_3_^−^ and NH_4_^+^ loadings and was therefore attributed to the secondary formation of ammonium nitrate aerosols. Cl is widely recognized as a distinct chemical tracer of coal combustion, indicating that this factor is closely associated with coal burning. Moreover, the predominated fraction of EC3 in addition with OC2 and OC3 sub-fractions in this factor was consistent with power plant source samples that emitted at high combustion temperature, which inferred this factor could be coal combustion that mainly from power plant and domestic heating boilers during wintertime. Overall, factor 4 was likely a mixed source influenced by nitrate-rich aerosols and coal combustion. The F4 was marked by elevated contributions of EC1 (37.4%), EC2 (33.4%), and OC2 (18.5%). This factor was mainly attributed to traffic emissions, as OC2 and EC1 are widely regarded as representative indicators of gasoline vehicle emissions, whereas EC2 was primarily linked to diesel vehicle emissions. Notably, this factor also exhibited high loadings of Zn (40.7%), Pb (50.2%), and Cu (39.4%). Zn is widely employed as an additive in engine lubricants, while Cu and Pb are typical constituents of brake wear emissions. The enrichment of these traffic-related trace metals further supports the attribution of this factor to vehicle emissions. The fifth factor was characterized by high loading of Ni (44.8%), Cr (25.0%), OC2 (25.0%), OC3 (24.0%), and EC1 (28.0%), which are closely related to industrial emissions from the industrial zone, which are characterized by petrochemical, non-ferrous metal industry and oil-refining facilities. The previous study reported that the carbon fractions (OC2 and OC3) emitted when burning coal at high temperature characterize. Thus, this factor was attributed to industrial dust in this study. The sixth factor (F6) was dominated by elevated contributions of Ni (44.3%), Cr (35.4%), Br (36.0%), and substantial contribution of SO_4_^2−^ (36.9%) and S (25.6%). These elements are closely related to urban waste incineration emissions around the sampling sites. Previous studies have shown that Cr and Ni are significantly enriched in high-temperature combustion processes such as waste incineration and can serve as characteristic tracers.^49^^,^^50^ In addition, the combustion of waste materials, particularly plastics and resins, can release various trace metals and halogen elements (e.g., Br), along with the formation of SO_4_^2−^.^51^

**Figure 5:**
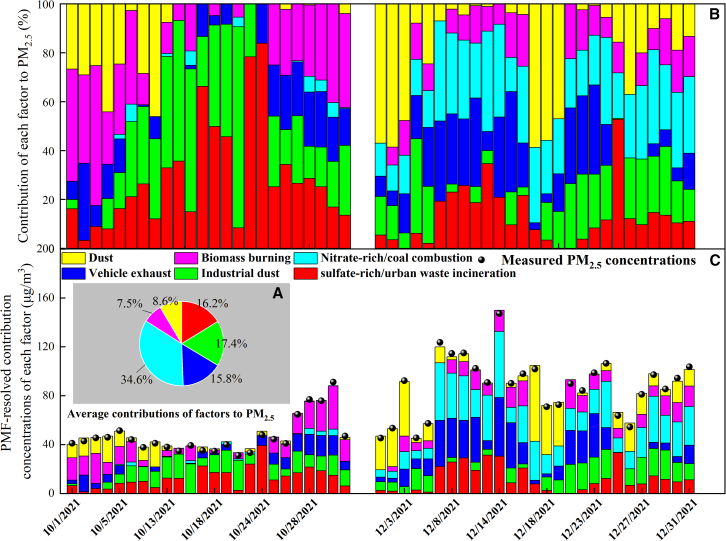
Source contributions of PMF-resolved factors in PM_2.5_ during the sampling period (A) Average contributions of each source. (B) Daily percentage contributions of each source. (C) Daily concentration contributions and comparison with measured PM_2.5_ mass.

Figure 5 illustrates the mean and day-to-day contributions of the PMF-resolved sources throughout the sampling period. Nitrate-rich/coal combustion, industrial dust, sulfate-rich/urban waste incineration, and vehicle exhaust were classified as the major contributors to PM_2.5_, accounting for 34.6%, 17.4%, 16.2%, and 15.8% of the total mass, respectively (Figure 5A). According to the daily contribution percentages in Figure 5B, dust, sulfate-rich/urban waste incineration, and industrial dust generally range between 15% and 45%. Among these, the contributions of sulfate-rich/urban waste incineration and industrial dust peak at up to 80% in autumn, while dust reaches up to 60% in winter, indicating that these sources are relatively stable primary contributors. No significant seasonal variation was observed for vehicle emissions, suggesting that these emissions at the suburban forest site exhibited regional characteristics during the study period. In comparison, the contribution from nitrate-rich and coal burning sources was significantly greater in winter than in autumn.

As shown in Figure 5C, the cumulative values of the stacked bars agree well with the measured PM_2.5_ concentrations (black dots), with over 85% of the PM_2.5_ mass explained, indicating that the PMF model successfully reproduces the majority of the total chemical species concentrations for each sample and confirming the robustness and reliability of the source apportionment despite the limited sample size (*n* = 51). Although the secondary sulfate factors were not clearly identified, significant quantities of sulfate were present in the coal burning and industrial emission factors. This suggests that some of these sulfate aerosols may have formed secondarily from gaseous precursors during their journey to the receptor site. Previous research has demonstrated that high temperature combustion promotes the oxidation of SO_2_ into sulfate, establishing coal burning as a significant a primary contributor to sulfate during winter heating seasons. Furthermore, given the relatively low SO_2_ concentrations (∼10 μg/m^3^) observed during the sampling period, secondary sulfate formation may have been limited to some extent. In contrast, the relatively high NO_2_ concentrations (∼60 μg/m^3^) suggest that nitrate aerosols were likely mainly formed through secondary processes. In addition, primary sulfate emissions from combustion sources, such as vehicle exhaust, could also contribute to ambient sulfate aerosols.

### Variations of δ^13^C and source implications for carbonaceous aerosols

#### Overall remarks on δ^13^C compositions

**Figure 6:**
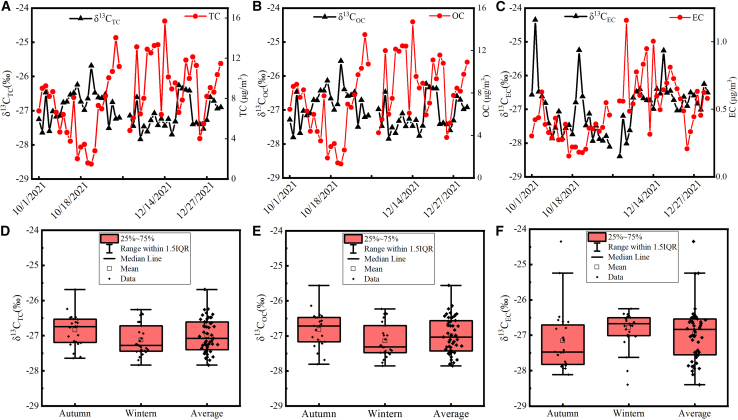
Stable carbon isotope compositions of carbonaceous aerosols at the suburban forest site (A) Temporal variations in δ^13^C_TC_ and TC concentration. (B) Temporal variations in δ^13^C_OC_ and OC concentration. (C) Temporal variations in δ^13^C_EC_ and EC concentration. (D) Seasonal box-and-whisker plots of δ^13^C_TC_. (E) Seasonal box-and-whisker plots of δ^13^C_OC_. (F) Seasonal box-and-whisker plots of δ^13^C_EC_.

The results above suggest that carbonaceous components accounted for a substantial proportion of PM_2.5_ at the suburban forest site, contributing up to 34.4% of the total mass. Figure 6 presents summary statistics of the levels of OC, EC, and TC, along with the stable carbon isotopic composition, at this suburban forest site. During the sampling period, the concentrations of OC, EC, and TC ranged from 1.31 to 14.70 μg/m^3^, 0.15 to 1.16 μg/m^3^, and 1.49 to 15.70 μg/m^3^, respectively.

**Figure 7:**
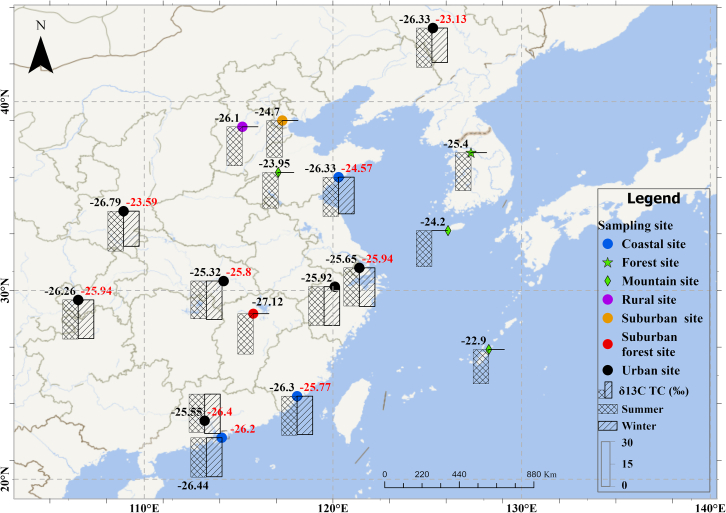
Spatial distribution of δ^13^C values (‰) of carbonaceous components (TC, and EC) at this suburban forest site and diverse atmospheric environments in the reported literature

The mean levels of OC, EC, and TC were 7.70 ± 3.30 μg/m^3^, 0.49 ± 0.22 μg/m^3^ and 8.19 ± 3.47 μg/m^3^. The carbonaceous fraction exhibited clear seasonal variability, with higher levels of TC, OC, and EC in PM_2.5_ during winter compared to autumn. The values of δ^13^C_TC_ at this suburban forest site varied from −27.83‰ to −25.69‰, with an average of −26.99‰ ± 0.47‰ during the sampling period. The δ^13^C TC value in winter (−27.12‰ ± 0.44‰) was slightly lower (i.e., more negative) than that in autumn aerosols (−26.84‰ ± 0.46‰) (t = −2.27, *p* = 0.028, independent-samples *t* test). While the δ^13^C_EC_ and δ^13^C_OC_ ranged from −27.85‰ to −25.56‰ (average −26.95‰ ± 0.76‰), and from −28.40‰ to −23.35‰ (average = −26.99‰ ± 0.52‰), respectively over the sampling period. The seasonal variations in δ^13^C_OC_ and δ^13^C_EC_ were consistent with those of δ^13^C_TC_, with relatively lower values in winter (i.e., more negative) and higher values in autumn (i.e., less negative). Compared to those of the reported δ^13^C_TC_ values for diverse atmospheric environments in the reported literature (Figure 7). The δ^13^C_TC_ average value observed at this suburban forest site was lighter than that at the background and remote sites, including costal sites, mountain sites and forest sites. For example, the δ^13^C value of fine-particle at this suburban forest site was ^13^C-depleted compared to the value (−23.68‰ ± 0.69‰) at mountain sites. It was also more negative than the measured values at the urban sites and suburban sites in northern Chinese cities. However, it was comparable to values reported for urban sites in southern Chinese cities in winter. For instance, it is lighter than the values observed in southern Chinese cities in winter (−26.0‰ ± 0.2‰)^21^ and northern Chinese cities (−25.6 ± 0.5‰).^52^ These variations can be explained by differences in pollution sources between suburban forests and other regions. An increase in δ^13^C indicates contributions from ^13^C-enriched sources to ambient aerosol loads. Notably, the variability of δ^13^C in TC at a given site is mainly influenced by regional and local scale burn sources, along with other human induced emissions. Urban sites are primarily affected by burning of fossil fuels and biomass. In contrast, biogenic releases and carbon transported over long distances are the primary influences on background and remote locations.

#### PSCF analysis

**Figure 8:**
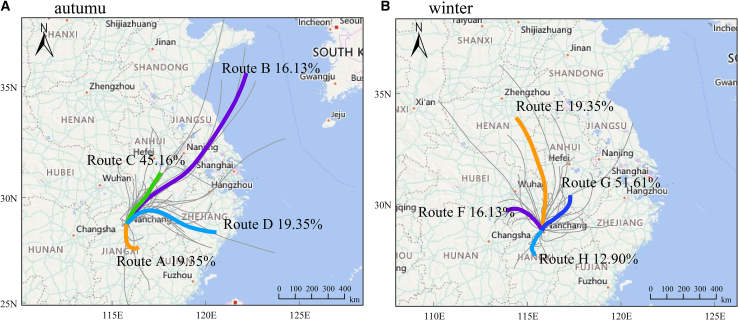
Cluster analysis of backward trajectories at the sampling site (A) Autumn. (B) Winter.

In this study, the HYSPLIT model was used to calculate 24 h backward trajectories for daily air masses during the sampling period, with the trajectory endpoint set at 900 m above ground level. The trajectories were further classified using cluster analysis for both autumn and winter (Figure 8). In autumn, four main transport pathways were identified. Route A exhibited the shortest transport distance and was considered a local emission pathway, with a contribution of 19.35%. In contrast, routes B, C, and D were associated with long-range transport, with contributions of 16.13%, 45.16%, and 19.35%, respectively. The contributions of the various sources to EC were fairly similar, and a similar pattern was observed for OC. Among them, route C showed the highest contribution, originating from south-central Anhui and passing through northern Jiangxi (e.g., Jiujiang) before reaching the sampling site. In contrast, route A showed a lower contribution and mainly originated from central Jiangxi, located to the south of the sampling site. In winter, four transport pathways were also identified. Routes E, F, and G were characterized by long-range transport, with contributions of 19.35%, 16.13%, and 51.61%, respectively, while route H represented a local emission pathway with the shortest transport distance and a contribution of 12.9%. The contributions of different pathways to EC were generally comparable. However, for OC, the local pathway (route H) showed the highest contribution.

**Figure 9:**
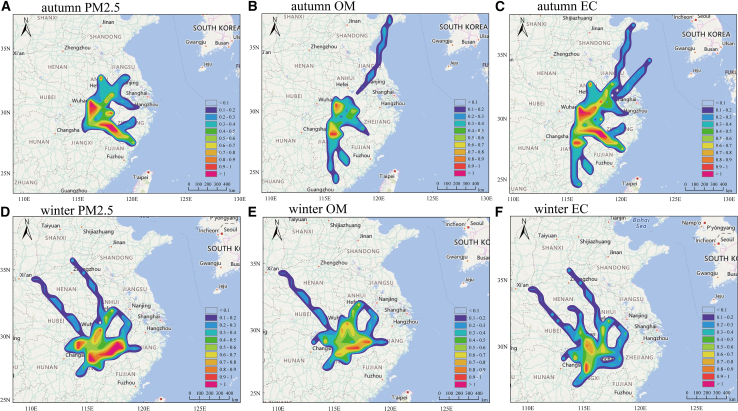
Spatial distributions of PSCF for PM_2.5_, OM, EC in PM_2.5_ at sampling site (A) Autumn PM_2.5_. (B) Autumn OM. (C) Autumn EC. (D) Winter PM_2.5_. (E) Winter OM. (F) Winter EC.

The PSCF results further identified the potential source regions contributing to PM_2.5_ and its components (Figure 9). In autumn, high PSCF values for PM_2.5_ and OC were mainly distributed in the border region between Hubei and Anhui provinces, as well as in the local area around Nanchang, indicating the combined influence of local emissions and regional transport. In contrast, high PSCF values for EC were more widely distributed and exhibited a distinct band-like pattern, reflecting a stronger influence of regional transport. Two major potential source regions were identified: one along the northeastern transport corridor associated with the Yangtze river delta region, including Nanjing (Jiangsu), southwestern Anhui, and Jiujiang (Jiangxi); and another mainly located within Jiangxi province, covering areas such as Shangrao, Jingdezhen, and Yingtan. In winter, high PSCF values for PM_2.5_ and OC were mainly concentrated in local and near-source regions, primarily distributed within Jiangxi Province, including Nanchang, Yingtan, and Shangrao, forming a northeast-oriented band. Similarly, EC was also dominated by local or near-source emissions, with major contributions from Xinyu in the southwest and Jiujiang in the north. These results highlight clear seasonal differences in source contributions, with PM_2.5_ and OC influenced by both local emissions and regional transport in autumn, whereas local sources play a more dominant role in winter.

#### Source identification of δ^13^C and impact of atmospheric processes

In contrast to the PMF analysis in the previous section, which focuses on the overall sources of PM_2.5_, this section employs isotopic analysis to investigate the sources of carbonaceous aerosols, thereby providing complementary insight into aerosol source apportionment. The stable carbon isotope (δ^13^C) values in carbonaceous aerosols exhibited distinct signatures across different emission sources.^53^ In urban environments, fossil fuel (such as liquid fuels and coal), biomass, and their combustion products are potential sources of carbonaceous component in PM_2.5_, along with contributions from geological sources. Number of studies have documented the δ^13^C values of liquid fossil fuels are within a range of −25.5‰ ± 1.3‰.^54^ This includes diesel (−26.0‰ ± 0.5‰, −28‰ to −24‰) and petrol-related emissions (−26‰ to −20‰), including unleaded petrol (−24.2‰ ± 0.6‰), high-grade petrol (−27.4‰ ± 0.1‰), and regular-grade petrol (−28.6‰ ± 0.2‰).^54^^,^^55^ Gaseous fossil-derived fuels (i.e., emissions from natural gas combustion; −27‰ to −23‰) are markedly more depleted in ^13^C than liquid fuels such as gasoline and diesel.^55^ With regard to solid fuels, coal combustion sources −25‰ to −21‰ (δ^13^C TC = −23.4‰ ± 1.3‰) can also contribute to TC fraction in aerosols.^56^ Emissions from the burn of C3 plants, such as trees, rice, and teak, have been reported to exhibit δ^13^C values ranging from −29.6‰ to −25.9‰, with a mean of −26.7‰ ± 1.8‰.^54^

In contrast, the emissions from C4 plants such as corn burning are within the range of −14‰ ± 0.7‰.^57^ Plants also produce VOCs, which contribute to the composition of the TC fraction. The δ^13^C analysis of α-pinene and β-pinene in carbonaceous aerosol varies between −29.5‰ and −28.4‰^58^ and −29.6‰ ± 0.2‰,^59^ respectively. The δ^13^C values of marine aerosols, including those from phytoplankton, ranges from −22‰ to −18‰, with an average of −21.0‰ ± 1.9‰.^60^ Besides the aforementioned sources, geological sources may also contribute to TC. The δ^13^C values of soil dust, street dust and rural soils are −10.5‰ ± 4.0‰,^61^ −17.0‰ ± 0.1‰ and −20.7‰ ± 1.5‰, respectively.^62^

**Figure 10:**
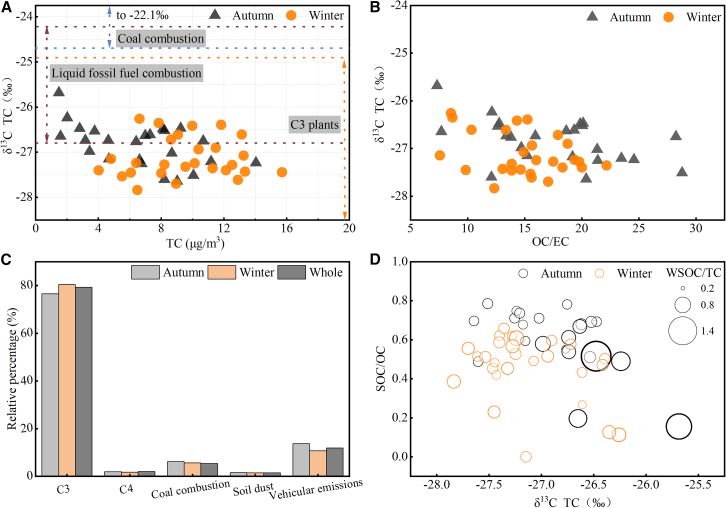
Source identification and atmospheric transformation of carbonaceous aerosols based on stable carbon isotopes (A) δ^13^C values of TC vs. the TC concentrations observed at this suburban forest site for the sampled seasons along with δ^13^C of sources (average as well as range) for source identification. (B) Plot of δ^13^C vs. EC/OC for the sampled seasons. (C) Proportional contribution of carbonaceous aerosol from various source-emissions by Bayesian model. (D) The scatterplot between SOC/OC and δ^13^C TC. The size of the bubble represents WSOC/TC.

Figure 10A shows δ^13^C_TC_ values of the particles released from various sources, as reported in the literature, together with those found at this suburban forest site during both autumn and winter. The δ^13^C distributions of PM_2.5_ at this suburban forest site were found to overlap with those of the C3 plant sources and liquid fossil combustion sources. These δ^13^C values were also compared with the source-specific OC/EC ratios to determine the dominant sources at the sampling sites (see Figure 10B). Earlier research has shown that the OC/EC ratios for petrol and diesel vehicle emissions range from 1.0 to 4.2, while coal combustion exhibits OC/EC ratios of 2.5–10.5. Moreover, ratios between 8.1 and 12.7 suggest contributions from biomass burning. These comparisons indicate that biomass burning (C3 plants), biogenic emissions, and gasoline vehicle exhaust are the predominant sources of atmospheric aerosols at this suburban forest site, although contributions from other sources cannot be excluded. In addition, a Bayesian model was applied to estimate the relative contributions of carbonaceous aerosols from multiple source emissions, including C3 and C4 plant, coal, soil dust, and liquid fossil fuels. Previous studies have demonstrated that Bayesian models can effectively quantify uncertainties in the source apportionment of carbonaceous aerosols^63^^,^^64^^,^^65^ and, in some cases, provide more robust estimates compared to traditional approaches such as PMF.^66^ The δ^13^C endmember values for each source were obtained from published literature and served as input for the Simmr model in this study. These sources comprise particles originating from, coal brun (δ^13^ C_coal_, −23.4‰ ± 1.3‰), vehicular emissions (δ^13^C_vehicle_, −25.5‰ ± 1.3‰), C3 plants (δ^13^C_C3 biomass_, −26.7‰ ± 1.8‰), C4 plants (δ^13^C_C3 biomass_, −14‰ ± 0.7‰) and soil dust (δ^13^C, −10.5‰ ± 4.0‰). The use of literature-derived δ^13^C end-member values may introduce uncertainty, as they may not fully represent the isotopic signatures of local emission sources. The carbonaceous components of PM_2.5_ in this suburban forest site mainly came from C3 plant biomass, vehicular emission, C4 plant biomass, coal, and soil dust, accounting for 79.3%, 11.9%, 2.0%, 5.4%, and 1.4%, respectively (Figure 10C).

The initial δ^13^C values are primarily determined by emission sources, whereas subsequent depletion or enrichment reflects atmospheric processing.^53^ Once aerosols are emitted, their δ^13^C signatures can be modified due to kinetic isotope effects (KIEs) during atmospheric processes, including SOC formation and photochemical aging. Oxidation of precursor gases via KIE generally results in lower δ^13^C values in SOC, while aging of aerosols leads to preferential loss of lighter carbon isotopes (e.g., CO and CO_2_), thereby enriching δ^13^C in the aged particles. During transport, organic aerosols experience oxidation and aging, which enhances their water solubility and leads to higher WSOC mass. Consequently, TC in freshly emitted aerosols contains a smaller fraction of WSOC than in transported aerosols, corresponding to higher WSOC/TC ratios during transport.

**Figure 11:**
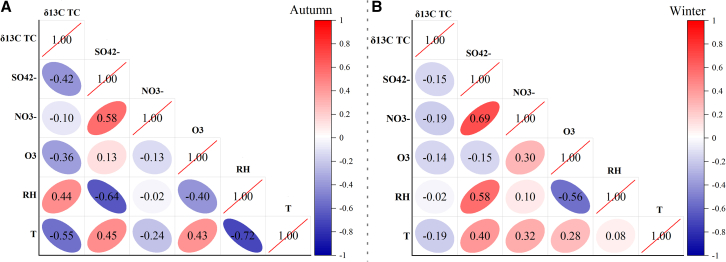
Correlation heatmaps of δ^13^C TC with gaseous pollutants and meteorological parameters during (A) The autumn season. (B) Winter season.

Therefore, δ^13^C, SOC and WSOC are used to understand the aging processes and formation of SOC from OC in the atmosphere. Our results showed that the WSOC/OC ratio at this suburban forest site varied between 0.2 and 0.94 (0.65 ± 0.16). The mean value ranged from 0.20 to 0.90 (0.58 ± 0.16) in autumn, and from 0.57 to 0.94 (0.75 ± 0.11) in winter. This indicates the presence of aged or SOC over at this suburban forest site, as the WSOC/OC ratio for primary biomass burning aerosols is around 0.50.^67^ Our results revealed negative correlations between δ^13^C_TC_ and SOC in autumn (r = - 0.51, *p* < 0.01) and winter (r = –0.81, *p* < 0.05), indicating that SOC formation leads to ^13^C depletion. Singh et al. also found that the δ^13^C values of aerosols in the Indian subcontinent shift toward more negative values as SOC content increases after the post-monsoon season,^68^ which is consistent with our findings and indicates that SOC formation influences the carbon isotope composition. A significant correlation was also observed between WSOC/TC and δ^13^C_TC_ at this suburban forest site during autumn (r = 0.52, *p* < 0.05), whereas no correlation was found during winter (r = 0.04, *p* > 0.05). Figure 10D shows the impact of the SOC formation and aging processes on δ^13^C during autumn and winter, combining the signatures of SOC/OC and WSOC/TC. More negative δ^13^C values were observed in winter, accompanied by lower WSOC/TC ratios and higher SOC/OC ratios. This may be due to the notable formation of fresh SOC in winter at this suburban forest site, which is located within a forest-dominated area (a source of biogenic VOCs). More positive δ^13^C values were observed in autumn, accompanied by lower WSOC/TC ratios and SOC/OC ratios. These results indicate that δ^13^C variations are not solely controlled by SOC formation, but reflect the combined effects of SOC formation and atmospheric aging. At this suburban forest site, δ^13^C values in autumn are influenced by both oxidation (i.e., aging) during the transport of air masses from urban areas and concurrent secondary formation processes. However, the relative importance of these processes differs. In autumn, aging processes dominate despite the higher SOC contribution, leading to enrichment of δ^13^C. In contrast, SOC formation plays a more important role in winter, resulting in more negative δ^13^C values. Therefore, δ^13^C_TC_ in suburban aerosols is ultimately governed by the relative contributions of oxidation and secondary formation processes. The δ^13^C value of TC in the particles can be impacted by the meteorological conditions and polluted precursor compounds, which can lead to alterations in the chemical processes. Studies have found that the suburban forest environments potentially have high RH, which is affected by both biogenic and anthropogenic emissions from surrounding urban areas with high levels of pollutants.^69^ Therefore, this study analyzed the correlations between δ^13^C values of TC at the suburban forest site during sampling and meteorological parameters (RH and T), the ambient oxidants (O_3_) and ion components (NO_3_^−^ and SO_4_^2−^). As shown in Figure 11, negative correlations were found between δ^13^C_TC_ and SO_4_^2−^, O_3_ and T in autumn, while positive correlations were found between δ^13^C_TC_ and RH. In winter, negative correlations were also found between δ^13^C_TC_ and meteorological parameters (RH and T) and ambient oxidants (O_3_), as well as between δ^13^C_TC_ and ion components (NO_3_^−^ and SO_4_^2−^), although these correlations were not strong. In general, SOC is generated through the physicochemical transformation of volatile precursors into the particle phase, either via photochemical oxidation in the atmosphere or through direct aqueous and heterogeneous reactions. For instance, VOCs emitted from both biogenic and human activities undergo atmospheric oxidation by ambient oxidants, leading to the formation of SOC and lower δ^13^C values in carbonaceous particles. Consequently, we found a negative correlation between O_3_ and δ^13^C values. However, the relative importance of these pathways varies with season. In winter, the lack of a significant correlation between δ^13^C and O_3_ suggests that SOC formation is not dominated by photochemical oxidation, but is more likely driven by aqueous-phase or heterogeneous processes.

On the other hand, the local RH levels are high during the autumn season (ranging from 52% to 90%, with an average of 70%) and during the winter season (ranging from 35% to 82%, with an average of 62%). At this suburban forest site, the aqueous phase formation gas-to-particle transformation predominates over gas-to-particle transformation. Also, both aging process and secondary formation processes occur simultaneously during the autumn season. Consequently, we observe a positive correlation between RH and δ^13^C values. This suggests that under high RH conditions, the isotopic enrichment associated with aging (e.g., heterogeneous oxidation and preferential loss of lighter ^12^C) outweighs the depletion effect caused by aqueous-phase SOC formation. Inorganic species have been shown to significantly facilitate the conversion of oxygenated compounds into organic phases, thus contributing to SOC formation.^70^ In this study, δ^13^C values exhibited a negative correlation with secondary inorganic species, including NO_3_^−^ and SO_4_^2−^.

## Summary and conclusions

This study presents the chemical and δ^13^C composition in PM_2.5_ at a suburban forest site in southern China during the autumn and winter seasons. The daily average level of PM_2.5_ varied between 31.14 and 147.16 μg/m^3^ (average 62.72 ± 28.90 μg/m^3^). Regarding individual chemical species, OM accounted for 19 ± 6% of PM_2.5_ mass, which was higher than the contributions of NO_3_^−^ (13% ± 8%), SO_4_^2−^ (9% ± 4%), CM (10% ± 4%), NH_4_^+^ (7% ± 2%), and EC (1% ± 0.1%). Secondary components and OM were the dominant components of PM_2.5_. The OC/EC ratio ranged between 6.19 and 28.79, with a mean value of 16.08, indicating that SOC was formed in this suburban forest site during the sampling time. Secondary OC (SOC) ranged between 0.20 and 10.10 μg/m^3^, with a mean value concentration of 4.27 μg/m^3^ during the sampling time. Furthermore, SOC contributions to OC were 60.40% and 46.16% in autumn and winter, respectively. The results demonstrated that organic compounds are prevalent in ambient particles at this suburban forest site, with the majority being SOC. OM was the dominant component in winter, while nitrate contributed more than sulfate during severe haze events. PMF identified and quantified six major source factors for PM_2.5_ including dust, biomass burning, nitrate-rich + coal combustion, vehicle exhaust, industrial dust, and urban waste incineration emissions, accounting for 34.6%, 17.4%, 16.2%, and 15.8% respectively.

We further investigated the carbonaceous aerosol characteristics. During the sampling campaign, the levels of OC, EC, and TC in PM_2.5_ ranged from 1.31 to 14.70 μg/m^3^, 0.15 to 1.16 μg/m^3^, and 1.49 to 15.7 μg/m^3^, respectively. The values of δ^13^C_TC_ at this suburban forest site varied from −27.83‰ to −25.69‰, with an average of −26.99‰ ± 0.47‰ over the sampling period. While the δ^13^C_EC_ and δ^13^C_OC_ ranged from −27.85‰ to −25.56‰ (average −26.95‰ ± 0.76‰), and from −28.40‰ to −23.35‰ (average = −26.99‰ ± 0.52‰), respectively during the sampling period. Bayesian model showed that it was mainly derived from C3 plant biomass (79.3%), vehicular emission (11.9%), C4 plant biomass (2.0%), coal (5.4%), and soil dust (1.4%). The WSOC/OC ratio at this suburban forest site spanning from 0.20 to 0.94 (0.65 ± 0.16), which indicates the presence of SOC or aged over at this suburban forest site. Lower δ^13^C values were found in winter, with lower WSOC/TC ratios and higher SOC/OC ratios. This could be due to the significant formation of fresh SOC in winter at this suburban forest site, which is located in a forest area (a source of biogenic VOCs). Higher δ^13^C values, however, were found in autumn, with higher WSOC/TC ratios and SOC/OC ratios.

### Limitations of the study

Several limitations should be acknowledged in this study. The sampling campaign was conducted only during autumn and winter at a suburban forest site, which may not fully reflect the seasonal and spatial variability of carbonaceous aerosols in southern China. Future observations covering multiple seasons and regions would improve the representativeness of the results. In addition, this study mainly focused on bulk chemical compositions and isotope characteristics, while detailed molecular tracer analysis and real-time observations of SOC formation were not included. Future studies integrating molecular-level source markers, radiocarbon analysis, and high-time-resolution observations would further improve the understanding of aerosol aging processes and secondary formation mechanisms in suburban forest environments.

## Resource availability

### Lead contact

Further information and requests for resources should be directed to and will be fulfilled by the lead contact, Jiaoping Xing (jiaopingx@jxau.edu.cn).

### Materials availability

This study did not generate new unique reagents or materials.

### Data and code availability

• All raw data have been deposited at Mendeley at (https://data.mendeley.com/datasets/2w32gf8sb4/1) and are publicly available as of the date of publication.
• This study does not report the original code.
• Any additional information required to reanalyze the data reported in this study is available from the lead contact upon request.

## Acknowledgments

This work was supported by National Natural Science Foundation of China (grant no. 42065007), and Natural Science Foundation of Jiangxi Province (20242BAB25388). We express sincere thanks to the Department of Jiangxi Provincial Key Laboratory of Conservation Biology (2023SSY02081) for their support during the project implementation.

## Author contributions

Conceptualization, data curation, formal analysis, investigation, methodology, software, and writing – original draft, Z.C.; data curation, formal analysis, and validation, Q.M.; conceptualization, funding acquisition, project administration, resources, supervision, and writing – review and editing, J.X.; software and validation, J.P.; investigation, methodology, and software, R.D.; investigation and methodology, F.C.; resources and supervision, W.W.; project administration, resources and supervision, Q.Y.; resources, Y. L.; resources and supervision, Y.W.; funding acquisition, project administration, and resources, L.Z.

## Declaration of interests

The authors declare no competing interests.

## STAR★Methods

### Key resources table

**Table:** 

REAGENT or RESOURCE	SOURCE	IDENTIFIER
**Chemicals, peptides, and recombinant proteins**		

Quartz fiber filters	Pall, USA	Tissuquartz-2500QAT-UP
High-volume sampler	Qingdao Laoshan Electronic Instrument Research Institute, China	KC-1000
Ion chromatograph	Dionex, USA	ICS-600
OC/EC analyzer	Sunset Laboratory, USA	Model 5 L
TOC analyzer	Analytik Jena, Germany	Multi N/C 2100

**Software and algorithms**		

EA-IRMS	Thermo Fisher Scientific, USA	Flash 2000HT
PMF model	US EPA	PMF 5.0
HYSPLIT model	NOAA	HYSPLIT
Trajectory analysis software	MeteoInfo	TrajStat plugin
Plotting software	OriginLab	Origin 2021

### Experimental model and study participant details

This study did not involve experimental models or study participants.

### Method details

#### Sample collection

This study involved collecting PM_2.5_ samples at a suburban forest site in Meiling National Forest Park in southern China between October and December 2021. The sampling site is surrounded by forest plants, farmland, and scattered residential areas, and is located approximately 3 km from the nearest highway and 11 km from the urban center (Figure 1). Sampling was conducted in autumn (October 2021) and winter (December 2021), yielding a total of 51 PM_2.5_ samples. The sampling instruments were installed on an open platform on the third floor, approximately 10 m above ground level. The samples were collected using a high-volume sampler (KC-1000, Qingdao Laoshan Electronic Instrument Research Institute, China). The sampling flow rate of 1050 L min^−1^, with a single sampling duration of 23.5 h. The sampling medium consisted of pretreated quartz fiber filter membranes (Tissuquartz-2500QAT-UP, 8 in × 10 in, Pall, USA), which were calcined at 450 °C for 4 h prior to sampling to eliminate residual organic matter. The PM_2.5_ mass concentration was determined using gravimetric analysis. Weighing was performed under constant temperature and humidity conditions using electronic balance (FA2004, Shanghai Precision Instrument Co., Ltd., China). After weighing, the filter membranes were sealed for storage and refrigerated at −18°C until subsequent chemical composition analysis was conducted.

#### Chemical analysis

##### Water-soluble inorganic ions

Water-soluble inorganic ions (Na^+^, NH_4_^+^, K^+^, Ca^2+^, Mg^2+^, F^−^, Cl^−^, NO_3_^−^and SO_4_^2−^) were measured using an ion chromatograph (Dionex ICS-600, USA). Two circular punches (16 mm in diameter) were taken from each PM_2.5_-loaded quartz filter, cut into small fragments, and placed in polyethylene bottles containing 20 mL of ultrapure water. The samples were subsequently agitated and centrifuged to extract the soluble fraction. The resulting extracts were drawn using a disposable 5 mL syringe and filtered through a 0.22 μm filter to remove impurities. The filtered solutions were then directly analyzed by ion chromatography for ionic species quantification. Quality assurance and quality control (QA/QC) procedures, including blanks, laboratory blanks, and repeated analyses for method detection limits, were implemented throughout the analytical process. The method detection limits (MDLs) for water-soluble inorganic ions were: Na^+^ (0.041 μg/mL), NH_4_^+^ (0.013 μg/mL), K^+^ (0.032 μg/mL), Ca^2+^ (0.174 μg/mL), Mg^2+^ (0.022 μg/mL), F^−^ (0.048 μg/mL), Cl^−^ (0.048 μg/mL), NO_3_^−^ (0.077 μg/mL), and SO_4_^2−^ (0.018 μg/mL), with all calibration curves showing excellent linearity (R^2^ = 0.999).

##### OC and EC analysis

The concentrations of OC and EC were measured using an OC/EC analyzer (Model 5 L, USA) according to the thermal-optical transmittance (TOT) method recommended by the National Institute for Occupational Safety and Health (NIOSH). A 1.0 cm × 1.5 cm section of each filter was cut out for the determination of OC and EC concentrations. Before analysis, the instrument was calibrated by standard gas. Comprehensive QA/QC procedures were applied, including the analysis of field blanks, laboratory blanks, and repeated measurements for method detection limits. The method exhibited a detection limit of 0.2 μg C/cm^2^. Calibration with standard sucrose solutions showed that the difference between the measured and reference values was less than 5%. Furthermore, replicate analyses yielded relative percentage deviations below 10% for both OC and EC.

##### WSOC analysis

WSOC was quantified using a total organic carbon (TOC) analyzer (Multi N/C 2100, Analytik Jena, Germany). Prior to analysis, the samples were pretreated as follows: a circular punch with an area of 3.14 cm^2^ was uniformly taken from each collected quartz filter and placed into a quartz vial that had been at 450 °C for 6 h. Subsequently, 10 mL of ultrapure (UP) water was added. The quartz vials containing the samples were extracted for 1 h using ultrasonication followed by mechanical shaking. The resulting extracts were then filtered through a 0.45 μm filter to remove particulate matter and residual quartz fibers. Finally, the WSOC level was determined using the TOC analyzer. Quality assurance and quality control (QA/QC) procedures, including blanks and replicate analyses, were implemented to ensure data reliability. The method detection limit (MDL) for WSOC was 0.12 mg L^−1^, and the calibration curve showed good linearity (R^2^ > 0.9) The recovery of WSOC ranged from 98.0% to 105%, and the relative standard deviation (RSD) was 2.8%, indicating good accuracy and precision.

##### PMF analysis

Source analysis of PM_2.5_ at Meiling National Forest Park was performed using the Positive Matrix Factorization (PMF) model. The detailed principles, model configuration, and uncertainty treatment have been described in our previous study.^71^

##### Sample analysis for δ^13^C

δ^13^C_TC_ and δ^13^C_EC_ in carbonaceous aerosols were determined using an elemental analyzer coupled to an isotope ratio mass spectrometer (EA-IRMS; Thermo Fisher Flash 2000HT, USA). Prior to analysis, the samples were subjected to pretreatment to remove inorganic carbon (IC), such as carbonates, thereby minimizing its potential influence on the results. Specifically, the samples were treated with 12 mol L^−1^ HCl to remove carbonate-derived IC, followed by heating at 60 °C for 4 h to volatilize residual acid. For the determination of δ^13^C_EC_, a circular punch was taken from a uniformly loaded quartz filter and placed into a quartz bottle containing CuO and Pb. The quartz bottle was evacuated to 10^−3^ Pa, sealed, and heated at 375 °C for 3 h, followed by cooling temperature. The pretreatment process is effective in reducing the residual OC content to less than 2%. After removal of OC, the remaining sample was encapsulated in a tin capsule and introduced into the elemental analyzer via an automatic sampler. For the determination of δ^13^C_TC_ no pretreatment was applied; the punched filter sample was directly encapsulated in a tin capsule and analyzed. δ^13^C_OC_ values were subsequently derived from δ^13^C_TC_ and δ^13^C_EC_ according to the following equation:

(Equation 1) $$\delta^{13}\;C_{TC}\times TC=\delta^{13}\;C_{OC}\times OC+\delta^{13}\;C_{EC}\times EC$$

(Equation 2) TC=OC+EC

In this equation, OC and EC denote mass level (μg·m^−3^). δ^13^C_TC_, δ^13^C_OC_, and δ^13^C_EC_ represent the stable carbon isotopic compositions. Analytical accuracy was ensured through the use of certified reference materials. δ^13^C measurements were calibrated using USGS40 (δ^13^C = −26.39‰) and USGS41a (δ^13^C = +36.55‰). At least one reference standard was included in each batch of 13 samples, and replicate analyses of the standards indicated a precision better than 0.2‰.

##### Bayesian isotope mixing model

A Bayesian isotope mixing model (MixSIAR) was applied to quantify the source contributions of carbonaceous aerosols. Within the Bayesian framework, receptor samples are represented as mixtures of multiple sources with unknown proportions. The δ^13^C end-member values were selected from published studies on emission sources in southern China to ensure regional representativeness and were expressed as mean ± standard deviation to account for source variability.

Source contributions and their associated uncertainties were estimated by combining likelihood functions with prior distributions using Markov chain Monte Carlo (MCMC) simulations.^72^ Model outputs are expressed as posterior distributions, which represent the probability density of the parameters and provide a quantitative measure of uncertainty, with results reported within the 90% credible intervals. The R package SIAR enables full access to these posterior distributions, allowing the extraction of summary statistics (e.g., mean, median, and credible intervals) for source contributions.

##### Air backward trajectory cluster and PSCF analysis

This study employed the Hybrid Single-Particle Lagrangian Trajectory (HYSPLIT) model, developed by the National Oceanic and Atmospheric Administration (NOAA), to perform a backward trajectory clustering analysis on the sampling points. For more detailed information about this model, please visit its official website (https://www.arl.noaa.gov/hysplit/). The meteorological data used to calculate transport trajectories were obtained from the Global Data Assimilation System (GDAS) provided by the National Centers for Environmental Prediction (NCEP) (https://www.ncei.noaa.gov/). The starting altitude for trajectory calculations was set at 900 m above ground level. The sampling point coordinates are 115°47′ E, 28°46′ N, and the simulation periods were October and December 2021. Based on the HYSPLIT model, the Potential Source Contribution Factor (PSCF) method was used to determine the spatial distribution. The PSCF analysis was performed using the TrajStat plugin in MeteoInfo software.^73^ For more details, please refer to the official website (Introduction - MeteoInfo 3.6 Documentation).

### Quantification and statistical analysis

All quantitative and statistical analyses were performed using Microsoft Excel, Python, and Origin 2022. Graphs were created using Origin 2026 (Figures 2, 3, 4, 5, 6, 10, and 11), HYSPLIT model (Figures 8 and 9), and ArcGIS 10.8 (Figures 1 and 7).
